# Where Are the Women in Radiation Oncology? A Cross-Sectional Multi-Specialty Comparative Analysis

**DOI:** 10.1016/j.adro.2021.100735

**Published:** 2021-06-04

**Authors:** Bismarck Odei, Jenna Kahn, Emma Brey Holliday, Dayssy Alexandra Diaz, Erika Bello-Pardo, James Odei, Junu Bae, Andrea Arnett, Raju Raval, Darrion Mitchell

**Affiliations:** a Ohio State University, Department of Radiation Oncology, Columbus, Ohio; b Oregon Health and Science University, Department of Radiation Oncology, Portland, Oregon; c MD Anderson Cancer Center, Department of Radiation Oncology, Houston, Texas; d Ohio State University, School of Public Health, Columbus, Ohio; e Ohio State University College of Public Health, Columbus, Ohio; f Ohio State University, School of Medicine, Columbus, Ohio

## Abstract

**Purpose:**

We aimed to evaluate the growth of women within the general radiation oncology (RO) workforce in comparison to the growth among other medical specialties. We also sought to create a predictive model for gender diversity to guide future recruitment efforts.

**Methods and Materials:**

We identified 16 medical specialties, including RO, for analyses. We used data from the Association of American Colleges and assessed female representation at 4 time points (2006, 2011, 2016, and 2020). Additionally, we determined characteristics of medical specialties that were predictive of increased gender diversity. We performed univariate statistical analysis with linear regression to evaluate factors predictive of greater gender diversity among the medical specialties in our cohort.

**Results:**

The proportion of women within the represented specialties increased over time. Obstetrics/gynecology (14,750 [2006], 23,921 [2020]; 18.7% absolute growth) and dermatology (3568 [2006], 6329 [2020]; 15.1% absolute growth) experienced the highest absolute growth in female representation between 2006 and 2020. When assessing changes between various time points in RO, the absolute change in female physicians increased by 1.5% between 2006 and 2011, by 2.2% between 2011 and 2016, and by only 0.4% between 2016 and 2020, which was the lowest growth pattern relative to the other 15 specialties. Factors predictive of gender diversity among specialties were lower average step 1 scores (*P* = .0056), fewer years of training (*P* = .0078), fewer work hours (*P* = .046), the availability of a standard third year clerkship for a given specialty (*P* = .0061), and a high baseline number of female physicians within a specialty (*P* = .0078). Research activities (*P* = .099) and interest among matriculating medical students (*P* = .28) were not statistically significant.

**Conclusions:**

The percentage of women in RO lags behind other medical specialties and has been notably low in the last few years. Interventions that incorporate novel initiatives proposed within this study may accelerate current recruitment milestones.

## Introduction

At the conclusion of the 1981 academic year, only 25% of graduating medical students in the United States were women.^1^ However, in 2019, the matriculating class of medical students was composed of more women than men (50.5%).^2^ Despite closure of the gender gap among medical school matriculants, asymmetrical distribution of female residents and attending physicians across medical specialties persists.^3^ Several factors have likely influenced this trend, including personal preference, prior exposure to a specialty, and mentorship, among other considerations.

In radiation oncology (RO) and among other historically male-dominated specialties, there have been growing efforts to increase the presence of women in the physician workforce and to eliminate barriers to mentorship and leadership.^4^, ^5^, ^6^, ^7^, ^8^ With the exception of a few studies^9^^,^^10^ highlighting variance in gender distribution between RO and other specialties, the majority of studies addressing gender concerns and reparative interventions in RO have often focused solely on female underrepresentation in RO^11^ and have thus lacked the benefit of objective comparison with other medical specialties.

In this study, we seek to provide a contemporary evaluation of the growth of women within the RO workforce compared with other specialties between 2006 and 2020 and assess if promising gains in gender diversity have been achieved within RO.

## Methods and Materials

### Study data

This was an institutional review board exempt, cross-sectional study. We obtained workforce data from the Association of American Colleges on 16 of the largest medical specialties with varying proportions of female presence.^12^ The data encompassed physician gender, medical specialties, and the number of practicing physicians and were reported for 2006, 2011, 2016, and 2020. We used these time points as a surrogate for trends over time, akin to similar approaches in literature.^13^

Physician data used in this study included those in the following settings: office-based patient care, hospital-based resident, hospital-based physician staff, administrative staff, medical teaching, research, locum tenens, or a hybrid position.

To create a model to predict gender diversity among specialties, the authors evaluated characteristics of specialties (years of specialty training, weekly work hours, baseline gender distribution within specialties), parameters affecting residency acceptance (average step 1 scores and research activities), and factors influencing exposure to a medical specialty (standard third year clerkship and interest of matriculating medical schools).

We obtained data from the Association of American Colleges on standard third year clerkship rotations in medical schools, length of postmedical school training per specialty, average weekly work hours per specialty, and interest in a specific specialty by matriculating medical students. Finally, we obtained data from Charting Outcomes^14^ from 2020 on average step 1 scores per specialty and the number of research activities of accepted candidates.

We performed univariate statistical analysis to evaluate factors predictive of greater gender diversity among medical specialties in our cohort. Linear regression models were used to examine associations between covariates and outcome. In addition, *t* test for regression slope was conducted. A 2-sided *P* value < .05 represented statistical significance.

## Results

The total number of women in our cohort of 16 specialties (Table 1) stratified by the 4 time points were as follows: 2006 (146,344; 29.8%), 2011 (176,517; 33.5%), 2016 (203,169; 37.0%), and 2020 (224,859; 39.2%). Collectively, primary care specialties constituted the highest absolute number of women in our cohort over time**.**

**Table 1:** Distribution of women among medical specialties in 2006 and 2020

Specialties	Number of women (2006)	%	Number of women (2020)	%	Absolute difference
	n = 146,344		n = 224,859		(%)
Pediatrics	27,761	53.5	38,405	64	64.3
Obstetrics and gynecology	14,750	40.9	23,921	60	59.6
Dermatology	3568	35.8	6329	51	51
Psychiatry	11,760	31.3	14,570	40	40
Clinical and anatomic pathology	4362	31.3	4399	39	39
Family practice	26,922	31.1	46,575	42	42.3
Internal medicine	30,573	30.7	45,994	39	38.7
Radiation oncology	952	23.4	1451	28	27.5
Neurology	2621	21.8	4306	31	30.7
Anesthesiology	7969	21.5	10,901	26	25.9
Diagnostic radiology	4576	21.3	6813	27	27.2
Emergency medicine	5681	20.1	12,699	28	28.3
Ophthalmology	3025	16.6	5091	27	26.6
Otolaryngology	911	10	1778	18	18.3
Neurologic surgery	250	5.1	532	9.3	4.2
Orthopedic surgery	663	3.3	1095	5.8	5.8

Female representation among RO physicians was 23.4% in 2006 and 27.5% in 2020 (4.1% increase), which was tied for the second lowest absolute growth over time among the represented specialties. Obstetrics/gynecology (14,750 [2006], 23,921 [2020]; 18.7% absolute growth) and dermatology (3568 [2006], 6329 [2020]; 15.1% absolute growth) experienced the highest absolute growth in female representation over the study period.

**Fig. 1:**
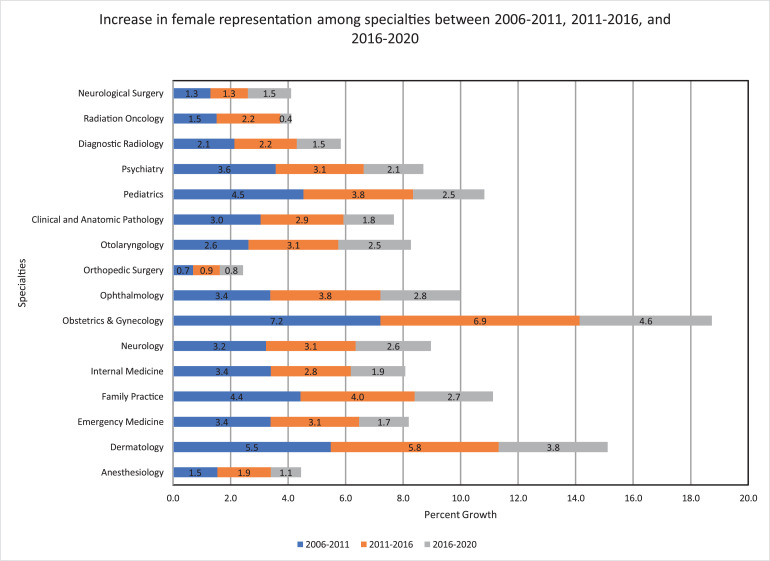
Growth of women physicians within several medical specialties at various time points.

When assessing changes between various time points in RO, the absolute change in female physicians increased by 1.5% between 2006 and 2011, by 2.2% between 2011 and 2016, and by only 0.4% between 2016 and 2020. The 0.4% increase between 2016 and 2020 was the lowest in comparison to the other 15 specialties (Figure 1).

Statistical analysis was undertaken on specialty characteristics (Table 2) and their association with gender diversity rank. Factors associated with a higher gender diversity rank were lower average step 1 scores (*P* = .0056), fewer years of training (*P* = .0078), fewer work hours (*P* = .046), the availability of a standard third year clerkship for a given specialty (*P* = .0061), and a high baseline number of female physicians within a specialty (*P* = .0078).

**Table 2:** Characteristics of medical specialties

Specialties	Gender diversity rank	Step 1 averagescore	Average number of research activities	Length of training	Average weekly work hours	Interest in specialty among matriculating medical students	Dedicated 3rd year clerkship	Number of active female physicians
				(years)				2020
Pediatrics	1st	228	4.9	3	47	9.00%	Yes	38,405
Obstetrics and gynecology	2nd	232	6	4	58	6.40%	Yes	23,921
Dermatology	3rd	248	19	4	45.4	2.80%	No	6329
Family practice	4th	221	3.3	3	52.6	5.00%	Yes	46,575
Psychiatry	5th	227	5.6	4	46.5	2.80%	Yes	21,827
Pathology	6th	233	7.3	4	*	0.60%	No	4399
Internal medicine	7th	235	6.2	3	54.9	13.10%	Yes	45,994
Neurology	8th	232	7.2	4	50.8	2.70%	Yes	4306
Emergency medicine	9th	233	4.3	3	46.4	8.60%	Sometimes	12,699
Radiation oncology	10th	243	18.3	5	51.8	1.00%	No	1451
Diagnostic radiology	11th	241	6.4	5	58	1.60%	No	6813
Ophthalmology	12th	245	*	3	51	2.10%	No	5091
Anesthesiology	13th	234	5.2	4	61	2.30%	Sometimes	10,901
Otolaryngology	14th	248	13.7	5	53.1	1.30%	No	1778
Neurologic surgery	15th	248	23.4	7	58.2	2.70%%	No	532
Orthopedic surgery	16th	248	14	5	57	7.20%	No	1095

⁎ Data are unavailable.

A low number of research activities (*P* = .099) and greater interest among matriculating medical students (*P* = .28) were also associated with higher gender diversity rank but were not statistically significant.

## Discussion

This study reports on the representation of women in RO in comparison to 15 other medical specialties between 2006 and 2020 and notes minimal increase in female representation in RO over time, with the most recent years showing an unprecedented low growth pattern relative to other specialties. Significant factors predictive of increased gender representation within a specialty were a lower step 1 average, fewer years of training, fewer work hours, availability of a standard third year clerkship, and a high baseline number of female physicians within a specialty.

Although our study evaluated predictive factors of gender diversity among all physicians (academic and nonacademic physicians), a previous study by Chapman et al^15^ reported predictive factors of gender diversity among specialties within academic medicine alone and also noted that a lower step 1 average, availability of a third year clerkship, and a high percentage of female faculty were predictive factors. Chapman et al, however, did not evaluate years of training and work hours as was done in the present study, but their study provides important preliminary findings that corroborate our results.

Our predictive model for intraspecialty diversity showed that high step 1 scores and research activities were not barriers to recruiting and matching women, as evidenced by the high proportion of women in dermatology. However, our results also suggest that specialties with an overemphasis on step 1 scores are generally less gender diverse. Consequently, efforts aimed at providing a more holistic approach to residency selection^16^ will augur well for gender diversity within RO.

We found that the inclusion of a specialty as part of the mandatory third year clerkship in medical school was predictive of increased gender diversity. The compounding realities that RO has low prematriculation interest (1%; Table 2) among medical students, limited inclusion in medical school curricula,^17^ and no standard third year clerkship may provide context for why achieving higher visibility among female medical students has likely been challenging. Moreover, the little exposure to RO during the early phase of medical school is further taxed by expectations of high research productivity among residency applicants,^14^ which may dissuade some female applicants who develop a late interest in RO from pursing the specialty. Thus, efforts targeting women during the premedical and preclerkship phases will be intrinsically important to effect change. National initiatives, under the auspices of the American Society for Radiation Oncology (ASTRO) and advanced by departmental leadership, which encourage engagement of undergraduate women as they obtain shadowing and clinical experience will be a valuable area of investment and will place the recruitment pipeline “more upstream.” Furthermore, a summer research fellowship that focuses exclusively on women early in their medical education may also be an important consideration.

Fewer years of training and fewer work hours were significant predictors of increased female representation within a specialty. The 5 years of postgraduate training in RO is longer than other specialties with a high female representation (obstetrics/gynecology [4 years], pediatrics [3 years], and dermatology [4 years]), suggesting that RO may be less attractive when considering training duration. However, the generally lower average weekly work hours in RO provides a favorable counterpoint, which may be underappreciated given the poor exposure of medical students to RO.

Our data also showed that a high baseline number of female physicians within a specialty is an important driver of female representation within a specialty and may reflect mentorship opportunities. Given the relatively small number of the female ROs in the workforce, and the likely geographic maldistribution of mentorship opportunities, a broader coalition of willing mentors will be needed to meet the current need for early female mentorship. It will be crucial for male ROs to enthusiastically seek opportunities to mentor women in the formative years of their medical training, especially in light of recent data showing the effectiveness of cross-gender mentorships.^18^ In this vein, current mentorship programs, including those sponsored by The Society for Women in Radiation Oncology,^19^ American College of Radiation Oncology,^20^ and The Gender Equity Community,^21^ require ongoing support and resources to achieve their important aims. Social media can also broaden the outreach of mentorship initiatives, particularly for female students at institutions without an affiliated RO department or in a scenario where only a few women mentors may be present. Ongoing efforts, such as the innovative #ILookLikeARadOnc and #WomenWhoCurie campaigns,^22^ will continue to be important in fostering inclusion and celebration of gender diversity within RO and will also be paramount in breaking down stereotypical gender barriers and perceptions.

Although female representation in the RO workforce has been historically modest, the factors specifically influencing the recent pattern of deceleration in female growth in RO are likely complex and multifactorial and may include concerns about the trajectory of the field. In spite of this recent trend, more efforts are present than ever before aimed at tackling the issue of improving gender diversity in RO.^23^ In June 2017 the board of directors of ASTRO approved a new strategic plan for RO,^24^ and among the 5 core values identified were “diversity” and “inclusion.” ASTRO's Committee of Health Equity, Diversity, and Inclusion also continues to work to raise awareness and improve the representation of women in RO. These efforts are laudable and the full effect of these initiatives may take time to be fully realized.

A limitation of our study is our use of 4 time points over time rather than reporting annual changes over time. We believe the time points functioned as strong surrogates of trends over time. Another limitation involves the use of limited specialty characteristics for our predictive model, which was likely not exhaustive of all possible factors to consider.

## Conclusions

The proportion of women in RO continues to lag behind other medical specialties, with a worsening trend in recent years. Effective change requires a commitment to steadfast and focused interventions that address the drivers of improved diversity, which include holistic residency admission processes, initiatives to increase exposure, and expansion of mentorship opportunities. Specifically, incorporating or expanding on initiatives such as a women's summer research fellowship, improved social media utilization, and early engagement of premedical students will be important. We call on stakeholders to explore these avenues to improve female representation in RO.

## Acknowledgments

We thank Dr Charles Thomas (OHSU) for his guidance during the course of this study.
